# Clinical Course of Cerebral Haemorrhage in Infective Endocarditis: Focus on Microbleed and Small-Area Haemorrhage

**DOI:** 10.1093/icvts/ivag154

**Published:** 2026-05-21

**Authors:** Yoshitaka Yamamoto, Masaki Ushijima, Ai Sakai, Yu Nosaka, Hiroki Nakabori, Hideyasu Ueda, Akira Murata, Kenji Iino

**Affiliations:** Department of Cardiovascular Surgery, Kanazawa University, Kanazawa 920-8641, Japan; Department of Cardiovascular Surgery, Kanazawa University, Kanazawa 920-8641, Japan; Department of Cardiovascular Surgery, Kanazawa University, Kanazawa 920-8641, Japan; Department of Cardiovascular Surgery, Kanazawa University, Kanazawa 920-8641, Japan; Department of Cardiovascular Surgery, Kanazawa University, Kanazawa 920-8641, Japan; Department of Cardiovascular Surgery, Kanazawa University, Kanazawa 920-8641, Japan; Department of Cardiovascular Surgery, Kanazawa University, Kanazawa 920-8641, Japan; Department of Cardiovascular Surgery, Kanazawa University, Kanazawa 920-8641, Japan

**Keywords:** infective endocarditis, intracranial haemorrhage, cerebral microbleed

## Abstract

**Objectives:**

This study aimed to review the surgical details and postoperative outcomes of on-pump valve surgery for preoperative central nervous system complications, especially infective endocarditis (IE) with cerebral microbleeds and small cerebral haemorrhage, in patients with acute IE.

**Methods:**

Between January 2015 and June 2024, we performed 54 surgeries for IE, with intracranial haemorrhage occurring in 13 of the 30 patients with preoperative central nervous system complications.

**Results:**

Among the 13 patients with intracranial haemorrhage, 11 had cerebral infarction, 6 had cerebral microbleed, 2 had small haemorrhages not meeting the definition of cerebral microbleed, and 5 had conventional haemorrhage. The median time from bleeding to surgery was 1, 2, and 20 days for cerebral microbleeds, small haemorrhages, and conventional haemorrhages, respectively. The main valve procedures were double valve replacement (5 patients), mitral valve replacement (6patients), and aortic valve replacement (2patients). Postoperative computed tomography (CT) showed worsening cerebral haemorrhage in 3 patients (23.1%, 95% CI, 5.0%-53.8%), including 1 with cerebral microbleed and 2 with small haemorrhage. Two patients with small haemorrhage required postoperative neurosurgical embolization (15.4%, 95% CI, 1.9%-45.4%). Postoperative complications included in-hospital death in 1 patient with conventional haemorrhage (7.7%, 95% CI, 0.2%-36.0%), but no deaths due to central nervous system complications were observed.

**Conclusions:**

In our cohort, over half of the patients with IE presented with central nervous system complications, with cerebral haemorrhage accounting for 43%. Postoperative haemorrhage worsened in all patients with small haemorrhage who underwent early intervention. Although the prognostic impact was minimal, small haemorrhages that were not cerebral microbleeds had a high rate of exacerbations requiring neurosurgery and should be treated with caution.

## Graphical abstract

**Figure:**
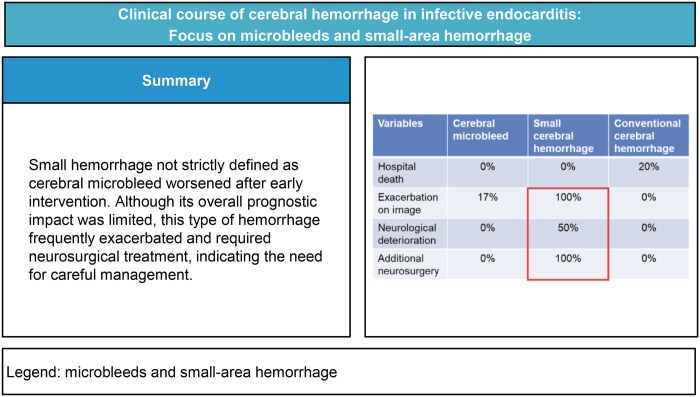

## INTRODUCTION

Neurological complications of various types and severity occur relatively commonly in infective endocarditis (IE). Despite certain opinions on the timing of surgical intervention for cerebral haemorrhage,^1^^,^^2^ it is often a difficult choice that depends on the condition of each individual. Although guidelines state that surgery should not be postponed for patients with cerebral microbleeds (CMBs),^3^^,^^4^ studies have reported postoperative cerebral haemorrhage exacerbation in such cases, which should warrant caution.^5^ Moreover, some cases fail to meet the strict definition of a microbleed but cannot be considered a major bleed, which complicates decisions regarding the timing of surgery. As neurological complications can affect both in-hospital and longer-term outcomes, it is clinically important to understand the postoperative risks associated with different types of haemorrhage. The present retrospective study, therefore, aimed to review our surgical experiences with IE cases complicated by cerebral haemorrhage and examine the differences in postoperative outcomes, including mortality, according to the type of haemorrhage.

## METHODS

### Patient selection

Data collection, analysis, and reporting were approved by the Institutional Review Board of Kanazawa University (reference No. 2024-167; approval date: 16 October 2024). Data on patients’ clinical characteristics, surgical data, and outcomes were obtained from the institutional surgical database. Written informed consent for the use of clinical data for research purposes was obtained from all patients at the time of admission. We identified 54 consecutive patients with infective endocarditis who underwent surgery at our institution between January 2015 and June 2024.

### Treatment strategy and operative procedures

The timing of surgery in each case was determined by the attending cardiologists and cardiac surgeons based on published guidelines. The surgical indications for IE were as follows: (1) acute decompensated heart failure, (2) uncontrolled infection, and (3) high embolic risk, including mobile vegetation.

The surgical procedure for IE consisted of complete debridement and repair or replacement of the infected structures, if needed. All patients underwent valve surgery under cardiopulmonary bypass (CPB) with systemic heparin administration for patients with an activated clotting time of >400 s. The body temperature during CPB was uniformly maintained at approximately 33 °C, corresponding to moderate hypothermia.

For valve replacement, the choice between a bioprosthetic and a mechanical valve was influenced by the patient’s age and, in younger patients, by the type of intracranial haemorrhage and the interval between the onset of intracranial haemorrhage (ICH) and surgery.

### Definitions

Infective endocarditis was defined as active if patients presented a fever or if positive blood cultures persisted despite adequate intravenous antibiotic therapy before surgery.

Magnetic resonance imaging was performed using standard-protocol diffusion-weighted imaging, fluid-attenuated inversion recovery, and magnetic resonance angiography. In addition, T2-star-weighted imaging or susceptibility-weighted imaging (SWI) sequences were used to assess CMBs, which were defined as hypointense lesions <10 mm in diameter on T2* or SWI. Hypointense lesions ≥10 mm in diameter were excluded from the cerebral microbleed (CMB) category. Lesions that did not meet the criteria for CMB were classified as conventional haemorrhage if they measured ≥10 mm on computed tomography (CT), or as small haemorrhage if they were not detectable on CT but measured ≥10 mm on MRI, or if they measured ≤10 mm on CT. For consistency, haemorrhagic lesions were categorized into 3 groups: CMB (CMB group), conventional haemorrhage (CH group), and small haemorrhage (SH group).

### Statistical analysis

Continuous variables were presented as the median and interquartile range, whereas categorical variables were presented as frequency and proportion. Categorical variables were compared using Fisher’s exact test. When all groups had 0 events, statistical comparison was considered not applicable because no variability existed across groups.

All statistical analyses were performed using EZR (Saitama Medical Center, Jichi Medical University, Saitama, Japan), which is a graphical user interface for R (The R Foundation for Statistical Computing, Vienna, Austria). More precisely, it is a modified version of R Commander designed to add statistical functions frequently used in biostatistics. The percentage values and 95% CI were calculated using the Clopper-Pearson exact method. Statistical inference was based on 2-tailed 0.05 thresholds, without multiplicity adjustments.

## RESULTS

### Baseline characteristics and perioperative data

**Figure 1.**
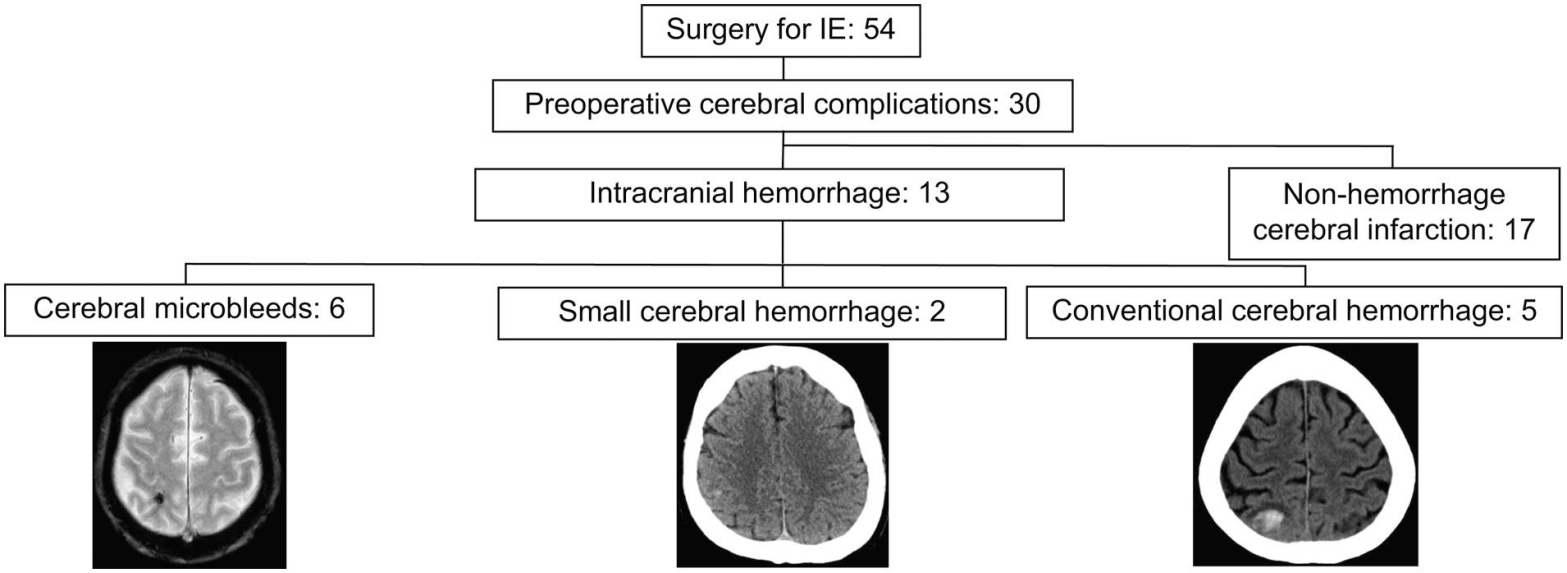
Flow Diagram of Study Enrollment. Representative Images for Each Group Are Shown at the Bottom of the Figure

Among the surgical cases of infective endocarditis included in this study, 30 (56%) were found to have central nervous system complications, among whom 13 (24%) were determined to have cerebral haemorrhage (**Figure 1**). The preoperative background information of the 13 (24%) patients with cerebral haemorrhage is summarized in **Table 1**. Magnetic resonance imaging (MRI) was performed in 12 (22%) patients. One (2%) patient who did not undergo MRI was diagnosed with subarachnoid haemorrhage based on computed tomography (CT) findings.

**Table 1.** Patients Background

Variables			Patients, *n* = 13	
Age (years), Median (IQR)			58.0 (48.0-67.0)	
Male, *n* (%)			4 (31)	
Diabetes, *n* (%)			0 (0)	
Hypertension, *n* (%)			5 (38)	
Hyperlipidaemia, *n* (%)			3 (23)	
Chronic kidney disease (eGFR <60 mL/min/1.73 m^2^), *n* (%)			5 (38)	
Haemodialysis, *n* (%)			2 (15)	
Reoperation, *n* (%)			2 (15)	
Steroid use, *n* (%)			1 (8)	
Type of endocarditis, *n* (%)	Native valve, first time	AV	11(85)	2(15)
		MV		5(38)
		AV + MV		4(31)
	Prosthetic valve (MV)		2 (15)	
Microbial aetiology, *n* (%)	Streptococcus		4 (31)	
	Staphylococcus		5 (38)	
	Others		4 (31)	
	Unknown		0 (0)	

Abbreviations: AV, aortic valve; eGFR, estimated glomerular filtration rate; IQR, interquartile range; .

Among the 12 patients who underwent MRI, 6 were found to have CMB, whereas 5 were diagnosed with conventional cerebral haemorrhage, although 2 of them were small haemorrhages that did not strictly meet the definition of CMB. Among the 2 patients with minor bleeding, one showed no bleeding on CT but revealed a low signal area ≥10 mm and evidence of very limited subarachnoid haemorrhage on the cortical surface of the left occipital lobe on T2* MRI sequence. In contrast, the other patient showed bleeding of 5 mm on CT. None of the patients exhibited a definitive cerebral aneurysm before surgery. A detailed comparison of CT and MRI findings in these 12 cases is summarized in **Table 2**.

**Table 2.** Comparison of CT and MRI Findings and Timing of Neurologic Deficit Onset in12 Patients

Patient no.	CT findings	MRI findings	Types of bleeding	Timing of neurologic deficit onset
1	Haemorrhagic infarction ≥10 mm	Haemorrhagic infarction ≥10 mm	CH	Stroke onset; haemorrhage + 2 months
2	Haemorrhage ≥10 mm	Haemorrhage ≥10 mm	CH	Stroke onset; haemorrhage + 3 days
3	No bleeding	Low-signal area ≥10 mm, very limited SAH on the cortical surface of the left occipital lobe on T2*	SH	None
4	No bleeding	Low-signal area <10 mm on T2*	CMBs	None
5	Haemorrhagic infarction ≥10 mm	N/A	CH	Stroke onset; haemorrhage + 3 days
6	No bleeding	Low-signal area <10 mm on T2*	CMBs	None
7	No bleeding	Low-signal area <10 mm on T2*	CMBs	None
8	5 mm haemorrhage	Low-signal area <10 mm on T2*	SH	None
9	No bleeding	Low-signal area <10 mm on T2*	CMBs	None
10	Haemorrhage ≥10 mm	Haemorrhage ≥10 mm	CH	Day 0
11	No bleeding	Low-signal area <10 mm on T2*	CMBs	None
12	No bleeding	Low-signal area <10 mm on T2*	CMBs	None

Patients are listed in chronological order of surgery.

For Patient No. 5, an MRI was performed only at the time of ischaemic stroke and not obtained during the haemorrhagic event.

Abbreviations: CH, conventional haemorrhage; CMBs, cerebral microbleeds; SH, small haemorrhage; SAH, subarachnoid haemorrhage.

**Table 3** summarizes the number of days until surgery, the surgical indication, and the operative status of 13 patients with cerebral haemorrhage. The median duration from the diagnosis of haemorrhage to surgical intervention was 1, 2, and 20 days in the CMB group, SH group, and CH group, respectively, indicating that surgical intervention was performed significantly earlier in the CMB and SH groups.

**Table 3.** Operative Timing and Indications for Surgery

Variables	Cerebral microbleed (*n* = 6)	Small cerebral haemorrhage (*n* = 2)	Conventional cerebral haemorrhage (*n* = 5)
**Time to surgery since diagnosis of ICH**			
Median (IQR)	1 (1-1)	2 (1.5-2.5)	20 (18-25)
<7 days	6	2	0
8-14 days	0	0	1
15-28 days	0	0	3
>28 days	0	0	1
**Time to surgery since diagnosis of IE**			
Median (IQR)	1.5 (1-2.75)	12 (7.5-16.5)	23 (20-23)
<7 days	5	1	0
8-14 days	0	0	0
15-28 days	0	1	4
>28 days	1	0	1
**Indications for surgery**			
Uncontrolled CHF	2	0	3
Uncontrolled infection	0	1	0
Mobile vegetation	4	1	2
**Operation status**			
Elective	0	0	1
Urgent	6	2	4
Emergent	0	0	0
Salvage	0	0	0

Abbreviations: CHF, congestive heart failure; ICH, intracranial haemorrhage; IE, infective endocarditis; IQR, interquartile range.

### Surgery and postoperative outcomes

The operative procedures used and postoperative outcomes are summarized in **Tables 4 and 5**, respectively. The major valve procedures included aortic valve replacement, double valve replacement, and mitral valve replacement, in 2, 5, and 6 patients, respectively.

**Table 4.** Operative Results

Variables			Cerebral microbleed (*n* = 6)	Small cerebral haemorrhage (*n *= 2)	Conventional cerebral haemorrhage (*n* = 5)	*P*-value
Single valve replacement	AVR	Biological	0 (0)	0 (0)	1 (20)	.538
		Mechanical	1 (17)	0 (0)	0 (0)	1
	MVR	Biological	2 (33)	1 (50)	2 (40)	1
		Mechanical	0 (0)	1 (50)	0 (0)	.154
Multiple valve (AV+MV) replacement	Biological		2 (33)	0 (0)	2 (40)	1
	Mechanical		1 (17)	0 (0)	0 (0)	1
Aortic cross-clamp time (min), Median (IQR)			121.5 (103.3-142.8)	76.0 (75.0-77.0)	137.0 (115.0-161.0)	.281
Cardiopulmonary bypass time (min), Median (IQR)			179.5 (137.3-213.5)	106.0 (101.0-111.0)	177.0 (169.0-249.0)	.281

Abbreviations: AV, aortic valve; AVR, aortic valve replacement; IQR, interquartile range; MV, mitral valve; MVR, mitral valve replacement.

**Table 5.** Postoperative Outcomes

Variables	Cerebral microbleed (*n* = 6)	Small cerebral haemorrhage (*n* = 2)	Conventional cerebral haemorrhage (*n* = 5)	*P*-value
Hospital deaths, *n* (%)	0 (0)	0 (0)	1 (20)	.538
Death due to neurological complication, *n* (%)	0 (0)	0 (0)	0 (0)	–
Exacerbation on image, *n* (%)	1 (17)	2 (100)	0 (0)	.0385
Neurological deterioration, *n* (%)	0 (0)	1 (50)	0 (0)	.154
Additional neurosurgery, *n* (%)	0 (0)	2 (100)	0 (0)	.0128

– Statistical comparison was not applicable because all groups had zero events for this variable.

**Figure 2.**
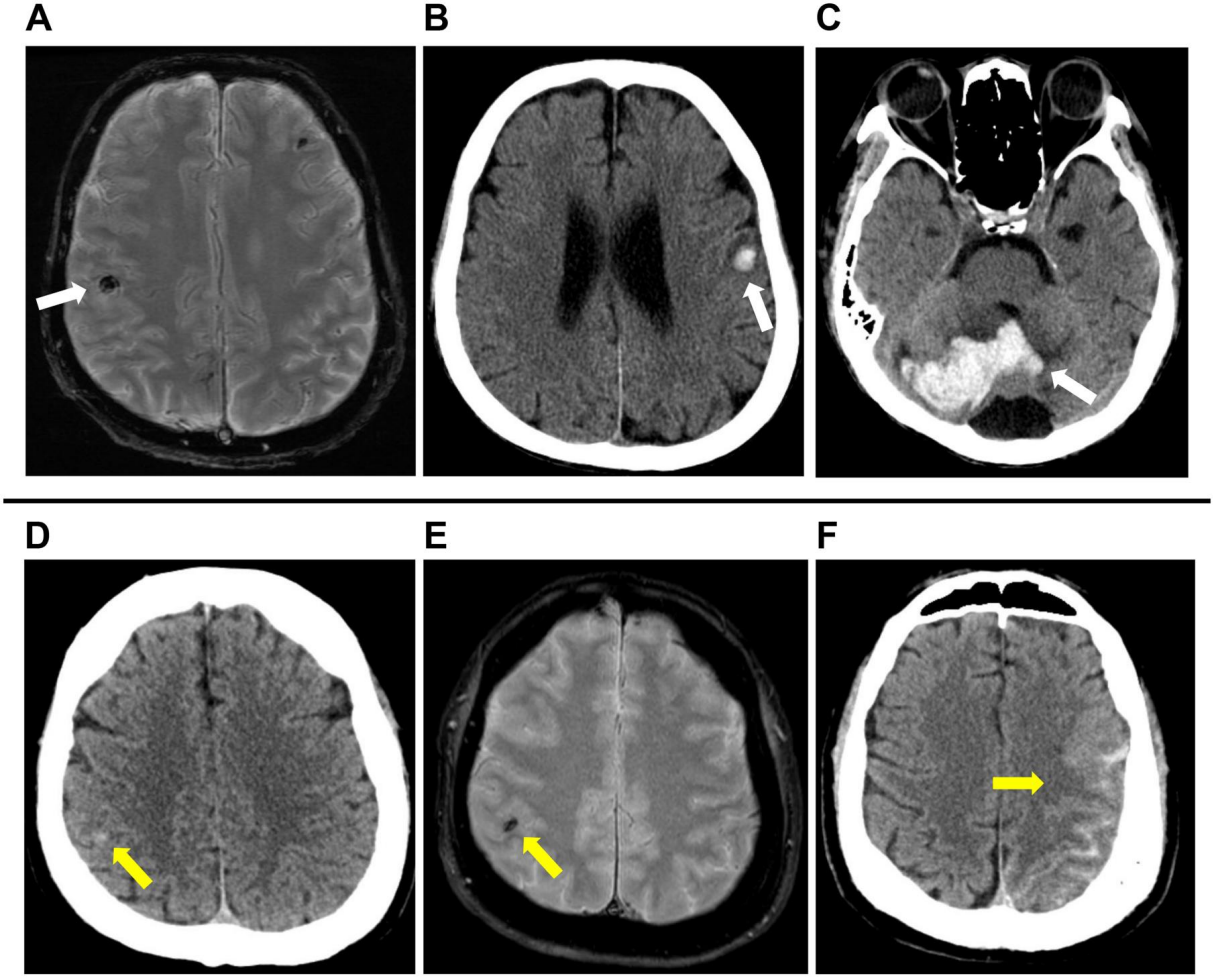
(A) Preoperative Magnetic Resonance Imaging of a Patient with a Small Haemorrhage. Computed Tomography (CT) Showed no Bleeding, but T2* MRI Sequence Revealed a Low Signal Area Measuring ≥10 mm. (B, C) CT on postoperative day 9 revealed cerebellar haemorrhage, subcortical bleeding in the left frontal lobe, and subarachnoid haemorrhage (SAH). (D, E) Preoperative CT and MRI of another patient with small haemorrhage showing bleeding with a diameter of 5 mm. (F) CT taken immediately after surgery revealed SAH

Postoperative cerebral haemorrhage was exacerbated in 3 patients (23.1%, 95% CI, 5.0%-53.8%). All of these cases underwent early surgical intervention for IE (within 3 days of cerebral haemorrhage diagnosis), including 2 patients with preoperative small haemorrhage and 1 patient with preoperative CMB. In 1 patient from the SH group (**Figure 2A**), nausea was observed on postoperative day 1. CT revealed cerebellar haemorrhage, subcortical bleeding in the left frontal lobe, and subarachnoid Haemorrhage (SAH) (**Figure 2B and C**). At that time, the source of the bleeding was unclear. However, a peripheral aneurysm in the right middle cerebral artery (MCA), which was located in a different region from the intracerebral haemorrhage, showed progressive enlargement over time, for which embolization was performed on postoperative day 9. For the second patient in the SH group (**Figure 2D and E**), CT performed immediately after surgery revealed SAH (**Figure 2F**). A peripheral aneurysm in the left MCA was identified, for which embolization was subsequently performed. In 1 patient with CMB, CT performed immediately after surgery revealed multiple haemorrhages, including one in the right basal ganglia. Owing to the small bleeding volume, a follow-up strategy with serial CT imaging was adopted. Among the patients with preoperative small haemorrhage, 2 required surgical intervention due to progressive bleeding, for whom endovascular aneurysm embolization was performed. In 1 of the 2 patients with small haemorrhage requiring neurosurgical management, neurological function declined on discharge, with the modified Rankin Scale increasing from 1 preoperatively to 4 at discharge. However, the patient was able to achieve social reintegration through postoperative rehabilitation. As shown in **Table 5**, the proportions of cases with imaging exacerbation and those requiring additional endovascular aneurysm embolization were significantly higher in the SH group compared with the other 2 groups (*P* = .0385 and *P* = .0128, respectively).

The overall in-hospital mortality rate, defined as death within 30 days or during initial hospitalization, was 7.7% (*n* = 1; 95% CI, 0.2%-36.0%). The cause of death was severe respiratory failure. In the overall sample, the rate of resternotomy for bleeding was 7.6% (*n* = 1), deep sternal infection occurred in 7.7% (*n* = 1; 95% CI, 0.2%-36.0%), and no deaths due to central nervous system-related complications were observed. The median follow-up duration was 268 days (IQR, 122-757 days).

## DISCUSSION

In patients with cerebral haemorrhage due to complications from IE, CMB, and small haemorrhages were treated with surgery at a relatively early stage, although some patients experienced worsening of postoperative cerebral haemorrhage. In particular, a high rate of endovascular aneurysm embolization was required for patients with small haemorrhage at our institution.

Central nervous system complications of various types and severity occur relatively commonly in patients with IE. Accordingly, current guidelines have provided consistent recommendations on the surgical treatment policy for severe cerebral infarction and cerebral haemorrhage. Regarding cerebral complications, the guidelines recommend waiting around 4 weeks, if possible, before surgical treatment for cerebral haemorrhage, except for microhaemorrhages.^3^^,^^4^

Brain MRI has excellent sensitivity in diagnosing acute ischaemic infarction, even in small or asymptomatic lesions. MRI can reveal asymptomatic, small, cerebral haemorrhages that are called CMB. CMBs, which correspond to iron deposits, have been recognized as markers of haemorrhagic change after thrombolysis, hypertensive vasculopathy, and cerebral amyloid angiopathy.^6^^,^^7^ Moreover, evidence suggests that CMB formation can predict the development of ICH during the clinical course of active IE.^8^ Although the pathophysiology of CMB formation in IE patients remains unclear, numerous investigators have hypothesized that CMB may represent a vascular vulnerability. The ESC guideline suggests that microbleeds do not warrant postponement of surgery when indicated, given the lack of an association between microbleeds and parenchymal haemorrhage and the absence of postoperative neurological complications.

At our facility, we also perform early surgery for patients with CMB, whereas for conventional haemorrhage we generally adopt a watchful waiting strategy as long as the patient’s condition allows. However, some patients present with small areas of bleeding lesions that do not meet the strict definition of a microbleed. Moreover, clearly distinguishing between CMB and small haemorrhages is often difficult; hence, early surgical intervention has been performed for both at our institution.

While early surgery for IE with cerebral bleeding has been increasingly advocated in recent years due to its survival benefits, it may carry a risk of adverse neurological outcomes.^1^^,^^2^ Although CMBs have been considered a potential risk factor for cerebral haemorrhage after cardiac surgery,^8^^,^^9^ recent findings suggest that their presence may not significantly affect mid-term or postoperative outcomes in patients with IE.^5^ Yoshioka *et al* concluded that valve surgery within 2 weeks of ICH onset may be relatively safe in patients with small haemorrhagic lesions considering that none of the 11 patients in their cohort experienced postoperative neurological deterioration. This study defined relatively small haemorrhagic lesions as small haemorrhages within infarctions, focal SAHs, or intracerebral haemorrhages 10 mm in diameter.^10^ Accordingly, 2 of our patients were determined to have small haemorrhages that did not fit the criteria for CMB. However, although they had no effect on survival after surgery, they did worsen to the point of requiring neurosurgical treatment. Although 1 patient achieved social reintegration through rehabilitation, neurological deterioration was observed.

Recent Japanese guidelines^11^ have also revised the recommendations for haemorrhagic cerebral complications in IE. While 4 weeks waiting period after haemorrhage was traditionally advised, the updated guideline acknowledges that many patients cannot tolerate such a delay due to haemodynamic instability, uncontrolled infection, or a high risk of recurrent embolism. Importantly, the guideline emphasizes assessing infectious aneurysms and the absence of haematoma expansion, and states that surgery within 2 weeks may be appropriate when these conditions are met. As the updated recommendations place particular emphasis on the absence of haematoma expansion when considering early surgery, it may be reasonable to incorporate short-interval imaging to confirm stability before proceeding with surgical intervention.

### Limitations

This study has some limitations worth noting. This was a nonrandomized, retrospective analysis conducted at a single center with a relatively small sample size. Consequently, differences in baseline characteristics among patients who underwent surgery for IE may have introduced bias into the results.

## CONCLUSION

Central nervous system complications, including cerebral infarction, were observed in 30 of the 56 patients at our institution, with intracerebral haemorrhage accounting for 43% of these cases. Among patients with small haemorrhages and CMBs who received early intervention, postoperative exacerbation of bleeding occurred in 38% (CMB group 17%, SH group 100%). Although small haemorrhages had a limited impact on overall prognosis, attention is warranted, as they tended to exacerbate after surgery and more frequently necessitated neurosurgical intervention.

## Data Availability

The data underlying this article cannot be made publicly available due to patient privacy and confidentiality restrictions. The data will be shared on a reasonable request to the corresponding author.

## Abbreviations

CMB: cerebral microbleed
CPB: cardiopulmonary bypass
CT: computed tomography
ICH: intracranial haemorrhage
IE: infective endocarditis
MRI: magnetic resonance imaging
SAH: subarachnoid haemorrhage
SWI: susceptibility-weighted imaging
